# Coupled Microwave/Photoassisted Methods for Environmental Remediation

**DOI:** 10.3390/molecules191118102

**Published:** 2014-11-05

**Authors:** Satoshi Horikoshi, Nick Serpone

**Affiliations:** 1 Department of Materials and Life Sciences, Faculty of Science and Technology, Sophia University, 7-1 Kioicho, Chiyodaku, Tokyo 102-8554, Japan; 2 PhotoGreen Laboratory, Dipartimento di Chimica, Università di Pavia, via Taramelli 12, Pavia 27100, Italy; E-Mail: nick.serpone@unipv.it

**Keywords:** photocatalyst, TiO_2_, microwave, non-thermal effect, wastewater treatment, microwave discharge electrodeless lamp

## Abstract

The microwave-induced acceleration of photocatalytic reactions was discovered serendipitously in the late 1990s. The activity of photocatalysts is enhanced significantly by both microwave radiation and UV light. Particularly relevant, other than as a heat source, was the enigmatic phenomenon of the non-thermal effect(s) of the microwave radiation that facilitated photocatalyzed reactions, as evidenced when examining various model contaminants in aqueous media. Results led to an examination of the possible mechanism(s) of the microwave effect(s). In the present article we contend that the microwaves’ non-thermal effect(s) is an important factor in the enhancement of TiO_2_-photoassisted reactions involving the decomposition of organic pollutants in model wastewaters by an integrated (coupled) microwave-/UV-illumination method (UV/MW). Moreover, such coupling of no less than two irradiation methods led to the fabrication and ultimate investigation of microwave discharged electrodeless lamps (MDELs) as optimal light sources; their use is also described. The review focuses on the enhanced activity of photocatalytic reactions when subjected to microwave radiation and concentrates on the authors’ research of the past few years.

## 1. Introduction

### 1.1. Microwave Radiation in Chemistry

Microwave radiation has become one of the more popular technologies, both domestically and industrially. It describes the low-energy electromagnetic radiation that spans the frequency range from 30 GHz to 300 MHz; that is, the wavelengths from 100 cm to 1 cm. Two rather familiar devices that make extensive use of this low-energy radiation are the domestic microwave oven and the cellular phone. Early (since 1949) industrial use of microwave radiation involved the thermal molding of wood and plastics, and the drying of medicinal products, fibers, teas, and cigarettes. In recent years, microwaves have been used in, among other applications, the sintering of ceramics, in cancer treatment (hyperthermia), in the drying and sterilization of foodstuffs, and in the vulcanization of rubber.

In the inorganic chemistry area, active research in the use of microwave radiation focused at the microwave sintering of ceramics (in the early 1980s) [1], in which the principal feature was the formation of compact crystal grains in a short time at relatively low temperatures. In the organic chemistry field, the use of microwave radiation to drive organic syntheses was not explored until the mid-1980s, at least not until the first two studies reported in 1986 by Gedye and coworkers [2] and by Giguere *et al.* [3] on microwave-enhanced organic processes using domestic microwave ovens. Since then, organic chemists have discovered the benefits of microwaves to drive synthetic reactions, as a consequence of which industries began to manufacture microwave ovens specifically designed for research laboratories. The number of reports on the use of microwaves as an energy source to drive chemical reactions has witnessed an astronomical growth since the early 1990s [4].

### 1.2. The Microwave-/Photo-Assisted Methodology

The photoassisted oxidative (and reductive) decomposition of pollutants by means of TiO_2_ semiconductor nanoparticulates is an effective and attractive oxidation (reduction) method in the general area of Advanced Oxidation Technologies. Several review articles have appeared that summarize environmental protection using TiO_2_ materials as the photomediators, if not as photocatalysts [5,6,7,8]. Applications of photoassisted treatments to air pollution have been developed by TiO_2_ fixation on such suitable substrate supports as filters in air conditioners, for instance [9]. However, this photoassisted degradation methodology is not suitable for large-scale wastewater treatment because the degradation rates of organic compounds dissolved in wastewaters tend to be rather slow. In this regard, relatively little has been done in this area in the last decade as large-scale treatments of organic pollutants in aquatic environments have not been without some problems, not least of which is the low photodegradation efficiency, a result of several factors, most notably: (i) the poor adsorption of wastewater organic pollutants on the TiO_2_ surface; (ii) the penetration of UV light tends to be shallow in turbid wastewaters; (iii) the need for dissolved oxygen in the photoassisted degradations; and (iv) the need to immobilize TiO_2_ nanoparticles in their use in aquatic ecosystems. Also relevant, the processing time has been the principal problem in actual wastewater treatments that have used TiO_2_ nanomaterials. Many of the above problems could be resolved if the activity of the photocatalysts were improved. To achieve such an objective we proposed some time ago [10] the coupling of both microwave radiation and ultraviolet radiation so as to enhance the activity of photocatalysts. With the latter coupled (*i.e.*, integrated) methodology, it was possible to enhance the photoassisted degradation processes in TiO_2_ dispersions by the added assistance of microwave radiation in the remediation of wastewaters contaminated with such pollutants as dyes, polymers, surfactants, herbicides, and endocrine disruptors, among others.

An integrated microwave-/photoassisted methodology presents certain advantages in wastewater treatment. In this technique, a feature of the reaction on the TiO_2_ surface involves thermal and specific effects (e.g., non-thermal effects) originating from the absorption of microwave radiation by the metal-oxide nanoparticulates. As a case in point, a microwave specific effect was inferred for P-25 TiO_2_ that exhibited lattice distortion and oxygen vacancies when used in UV-driven/microwave-assisted photocatalyzed reactions [11]. Differences between various TiO_2_ batches with regard to microwave-specific effect(s) were examined using microwaves of different frequencies [12], and by examining the effects of the microwaves’ magnetic and electric fields [13] in photoassisted processes involving TiO_2_ and ZnO nanomaterials. Increased formation of ^•^OH radicals on the surface of various TiO_2_ specimens that were exposed to microwave radiation correlated with increased photoactivity [11]. The microwave specific effect, not encountered when the photocatalyzed reaction is subjected to conventional heating, inferred that the composition and the electronic characteristics of the TiO_2_ nanomaterials were important factors.

In the present article, we review some of our research studies carried out during the past few years that have focused on improving the photocatalytic activity of TiO_2_ by exposing it to microwave radiation. The enhancement of the photoactivity of TiO_2_, in particular, and metal-oxide nanomaterials, in general, by microwave non-thermal effect(s) is discoursed in this review paper. We further describe the utility of microwave discharged electrodeless lamps (MDELs) as an optimal light source that embeds the integrated UV/MW technique.

## 2. Experimental Setup of an Integrated Microwave/Photoreactor System

**Figure 1:**
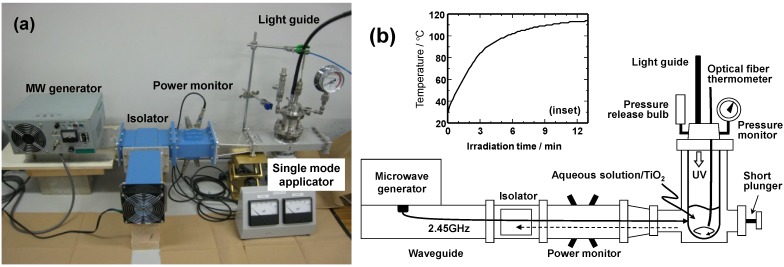
(**a**) Photograph of an integrated microwave/photoreactor system having a single-mode applicator, and (**b**) schematic of the system and a typical plot (inset) of the change of temperature with irradiation time for an aqueous TiO_2_ dispersion under microwave irradiation.

Continuous microwave irradiation of a wastewater sample can typically be achieved in a single-mode applicator using a 2.45-GHz microwave generator, a power monitor (to assess incident and reflected microwave power), a three stub tuner and an isolator (an air cooling device) such as the one fabricated by the Hitachi Kyowa Engineering Co. Ltd. (Hitachi, Ibaraki Prefecture, Japan; Figure 1a). A typical reactor setup might contain a model wastewater sample (30 mL) containing TiO_2_ particles (Evonik P25; 60 mg loading) introduced into a closed high-pressure 150-mL Pyrex glass cylindrical reactor. Subsequent irradiation of the reactor with UV light from a super high-pressure Hg lamp can be achieved through the means of a light guide. The solution temperature is normally measured with an optical fiber thermometer. A pressure gauge and a release bulb are also connected to the cover of the reactor. The reaction mixture is then stirred continually using a magnetic bar during the UV light irradiation or coupled UV/microwave (MW) irradiation.

Three different methodologies have typically been used to examine the photodecomposition of aqueous samples of pollutants in aqueous TiO_2_ dispersions. The first is the photo-/microwave-assisted method using UV light and microwave irradiation in the presence of TiO_2_ (UV/MW). The second method entails UV irradiation alone (UV), whereas the third method involves a thermally-assisted photodegradation of the TiO_2_ dispersions using UV light and externally applied conventional heat (UV/CH; typically from an oil bath or an electric heat mantle). In the present context, the external heat can be supplied by coating one part of the cylindrical photoreactor with a thin metallic film on one side at the bottom of the reactor, whereas the uncoated side permits the UV radiation to reach the dispersion. The pressure and the rate of increase of temperature (error typically ≤ ±1 °C) in the UV/CH method are maintained at levels otherwise identical to those used in the UV/MW methodology. As such, no differences in temperature profiles are observed when using either microwave dielectric heating or conventional heating.

## 3. Description and Discussion of Results from the Various Studies

### 3.1. Degradation of Substances Incompatible with TiO_2_ Photocatalysts

**Figure 2:**
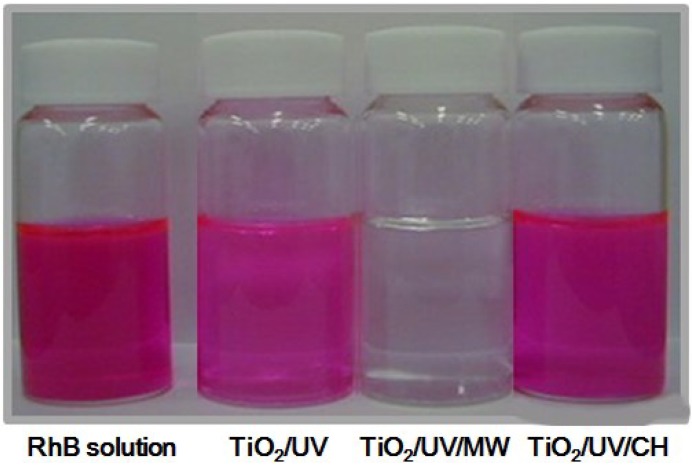
Visual comparison of color fading in the degradation of RhB solutions (0.05 mM) subsequent to being subjected to various degradation methods for 150 min. From left to right: initial RhB solution; RhB subjected to photoassisted degradation (UV); EhB subjected to integrated microwave-/photo-assisted degradation (UV/MW); RhN subjected to thermal- and photo-assisted degradation (UV/CH). Reproduced from [17]. Copyright 2009 by Elsevier B.V.

The photooxidative remediation of wastewaters with TiO_2_ as the photocatalyst has been reported in many international journals (see for example the references in [14]). In the degradation of a dye substrate, the photoassisted reaction can be accomplished by means of electron transfer from the excited dye that is exposed to visible light. In this regard, it is noteworthy that the Japanese Industrial Standards (JIS) includes the methylene blue dye as a standard wastewater substance to ascertain the activity of photocatalysts [15]. Some dyes are poorly photodecomposed and thus are not useful in ascertaining the photoactivity of a metal-oxide photocatalyst. For example, the rate of photodegradation of the cationic dye rhodamine-B (RhB) is slow in acidic aqueous media because the surface of TiO_2_ particles is positively charged (Ti-OH_2_^+^; pI = 6.3). However, RhB has proven as an interesting model compound to examine the microwave effect. In earlier studies, the major focus of our studies was on the degradation of organic pollutants, as exemplified by the degradation of the rhodamine-B (RhB) dye catalyzed by TiO_2_ semiconductor particles under both UV and microwave irradiation [10,16]. Changes in color intensity of the RhB dye solutions occurring under various conditions are illustrated in Figure 2. The photodegradation of RhB is clearly evident on using the TiO_2_-assisted UV/MW method. These observations demonstrate that a method that can treat large quantities of pollutants in wastewaters by a hybrid combination of microwaves and TiO_2_ photoassisted technologies is conceivable. The photodegradation by this metal oxide is unaffected by conventional heating (CH)—compare, for example, the results from the UV and the UV/CH methods in the presence of TiO_2_ (Figure 2) [17].

**Figure 3:**
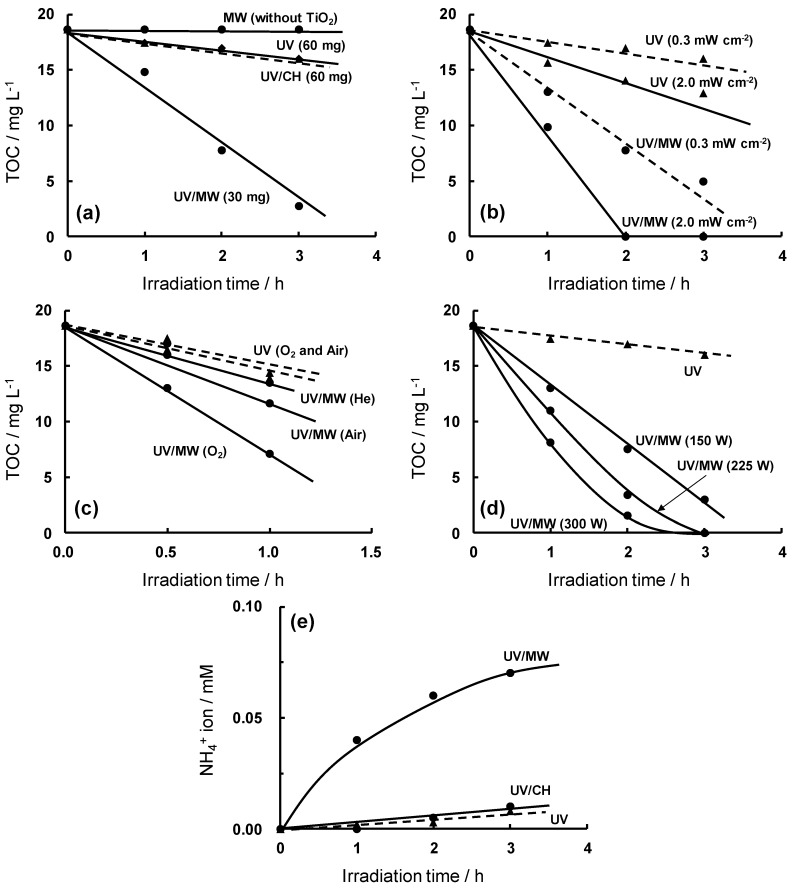
(**a**) Decrease of total organic carbon (TOC) in the decomposition of RhB solution (initial TOC concentration, 18.6 mg·L^−1^; 30 mL) by MW (without TiO_2_), UV (60 mg), UV/CH (60 mg) and UV/MW (30 mg); (**b**) Temporal evolution of the decrease of TOC during the degradation of RhB solution (0.050 mM, 30 mL) at a radiance of 0.3 and 2.0 mW·cm^−2^; (**c**) Decrease of TOC values for the influence of different added gases on the degradation of RhB (0.050 mM); (**d**) Decrease of TOC for RhB solutions (0.050 mM, 30 mL) with TiO_2_ loading (30 mg) by the UV/MW method (microwave applied power at 150 W, 225 W and 300 W); (**e**) Temporal evolution of the formation of NH_4_^+^ ions in the decomposition of RhB (0.050 mM) using the UV, UV/CH and UV/MW methods; the radiance was 0.3 mW·cm^−2^. Reproduced from [10]. Copyright 2002 by the American Chemical Society.

Microwave effects were examined by the temporal decay of total organic carbon (TOC) in the degradation of aqueous RhB solutions under four different methodologies. Results displayed in Figure 3a show that for a TiO_2_ loading of 60 mg (volume 30 mL) there is no distinction between the efficacy of the UV and UV/CH methods. In the absence of the metal oxide TiO_2_, no changes in TOC occurred when the RhB dye solution was irradiated only by the microwaves, even after 3 h. At a TiO_2_ loading of 30 mg (volume 30 mL) the UV/MW method proved very efficient in decreasing the TOC by nearly a factor of six from 18.6 mg·L^−1^ (ppm) to about 3 mg·L^−1^ after 3 h.

The degradation of RhB was also examined at two different UV-light irradiances (0.3 and 2.0 mW·cm^−2^) to assess the microwave effects through the loss of TOC as depicted in Figure 3b. Clearly, the degradation of RhB by the UV/ MW was faster even at the lower irradiance of 0.3 mW·cm^−2^ than occurred by the UV method at the higher irradiance of 2.0 mW·cm^−2^. Evidently, the situation at the lower UV-light irradiance accentuated the effect of the microwave radiation.

The disappearance of TOC in aqueous RhB solutions under various atmospheric conditions (air, oxygen gas, and helium gas) in the presence of TiO_2_ is illustrated in Figure 3c. Recombination of photogenerated valence band holes with conduction band electrons after UV irradiation of TiO_2_ particles is known to compete with formation of reactive oxygen species (^•^OH and ^•^OOH radicals) that typically lead to the degradative processes. The degradation of an organic pollutant via a TiO_2_ photoassisted reaction typically follows the order oxygen gas > air > inert gas (e.g., He). In the present instance, integrating microwave irradiation to UV irradiation for an oxygen-saturated solution led to twofold enhancement in the decrease of TOC [10]. Moreover, the decrease of TOC in the air-equilibrated RhB solution by the UV/MW method was enhanced relative to the degradation of oxygen-saturated RhB solutions by the UV method alone. Within experimental error, the decomposition rate of the helium-purged RhB solution by the UV/MW method was nearly the same as the rate for an oxygen-purged dye solution by the UV method alone, again indicating the influence of microwave factors in the degradation process.

The impact of microwave power on the degradation of RhB solutions is illustrated in Figure 3d. The temperature rise from ambient (25 °C) at power levels of 150, 225 and 300 W was 62, 75 and 138 °C, respectively, within the first 20 min of irradiation. The increase in microwave power enhanced the degradation of RhB solutions, as witnessed by the decrease of TOC for an irradiation period of about 2 h: the decrease in TOC by the UV/MW was 61% at a microwave power of 150 W, 82% for 225 W, and 92% at 300 W. In the absence of MW irradiation, the extent of TOC decrease by the UV method alone was only 14%. Clearly, under the conditions used, microwave power output enhanced the decomposition dynamics.

Formation of NH_4_^+^ (and NO_3_^−^) ions in the decomposition of RhB was also investigated by the UV, UV/CH and UV/MW methods in the presence of TiO_2_ nanoparticulates (only data of NH_4_^+^ are shown in Figure 3e) [10]. Again, the UV/MW method led to more significant changes than either the UV or UV/CH methods alone in converting the two nitrogen atoms in the RhB structure. In this regard, the mineralization yields of the two nitrogen atoms, given as the sum of the yields of NH_4_^+^ and NO_3_^−^ ions, respectively, after 3 h of irradiation were 77% (70% + 7%) for the UV/MW procedure, 12.8% (10% + 2.8%) for the UV method, and 8% (8% + 0%) for the UV/CH method. The increased formation yield of NH_4_^+^ ions by the UV/MW method was significant by comparison with the UV and UV/CH methods. These features inferred that the mechanistic details in the decomposition of RhB in aqueous media in the presence of TiO_2_ differed under UV/MW irradiation relative to the UV-induced degradation.

**Scheme 1:**
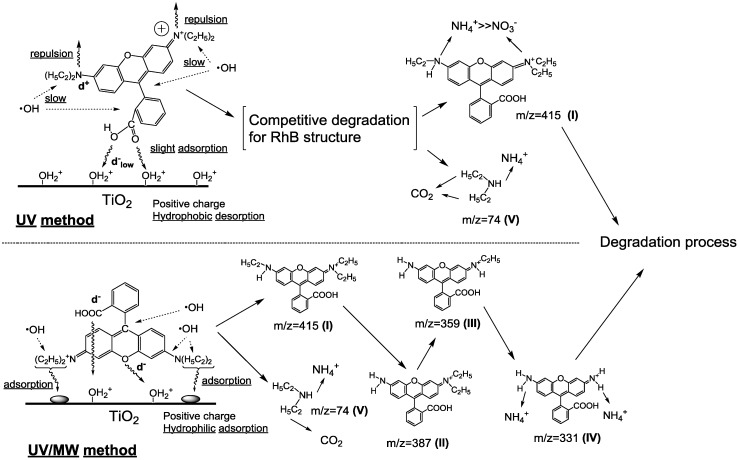
Proposed initial mechanistic steps in the degradation of RhB dye by the UV and UV/MW methods. Reproduced from [16]. Copyright 2002 by the American Chemical Society.

The initially-formed intermediates from the degraded RhB were identified by electrospray ESI ionization mass spectra (positive ion mode) and subsequently confirmed by HPLC/absorption spectroscopy [16]. An initial adsorption model of RhB molecule on the TiO_2_ surface was proposed by computer simulations that led to estimates of frontier electron densities of all atoms in the RhB structure, which afforded inferences as to the position of ^•^OH (or HOO^•^) radical attack on the RhB structure. Results from these simulations led to the proposed degradation mechanisms summarized in Scheme 1 [16]. For the UV method, RhB approaches the positively charged TiO_2_ surface through the two oxygen atoms in the carboxylate function bearing the greater negative charge, with further assistance provided by the repulsion between the positively charged nitrogen atoms and the positive TiO_2_ surface. Accordingly, de-ethylation of RhB by the UV method was rather inefficient as evidenced by the formation of intermediate (**I**) only (LC/MSD analysis).

Diethylamine was formed through the cleavage of the C-N bond in the C-N(C_2_H_5_)_2_ fragment of RhB. For the UV/MW method, the increase of the hydrophobic nature of TiO_2_ through microwave irradiation facilitated adsorption of RhB through the aromatic rings aided by the three oxygen atoms in RhB; that is, RhB lay flat on the particle surface. The principal intermediates formed in the degradation of RhB by the UV/MW method were the *N*-de-ethylated species **I** to **IV**. Ultimately, the amino groups of intermediate **IV** were converted predominantly into NH_4_^+^ ions. The hydrophobic methyl component of the ethyl group could not adsorb onto the hydrophilic TiO_2_ surface, so that transformation of the nitrogen atoms was not a priority event in the photoassisted process.

The surface electric charge of TiO_2_ particles is another important factor that impinges on the adsorption of substrates on the TiO_2_ surface. Accordingly, the electric charge on the TiO_2_ surface was ascertained by a zeta potential analysis using the coupled microwave/UV irradiation system [13]. Under UV light irradiation alone, the zeta-potential was positive in acidic media and decreased with increase in pH of the aqueous TiO_2_ dispersions. The point of zero charge (pzc) of the TiO_2_ particles under UV irradiation was attained at pH ~ 6.7. On the other hand, the typical zeta-potential curve was not evident under simultaneous UV/MW irradiation conditions. In the latter case, the zeta-potential remained somewhat positive in the 0–20 mV range for dispersions throughout the pH range 4–9. A change of the TiO_2_ surface charge by the microwave radiation changes the adsorbed state of RhB in a manner that facilitates the cationic nitrogen group to approach the TiO_2_ surface.

### 3.2. Degradation of 2,4-Dichlorophenoxyacetic Acid (2,4-D) in Aqueous TiO_2_ Dispersion

Many studies have reported the photocatalyzed decomposition of such chlorinated pollutants as the agrochemicals containing the triazine skeleton (atrazine) [18], dibromochloropropane [19], hexachlorocyclohexane [20], *p,p'*-DDT [21], *p,p'*-DDE [22], 2,4-dichlorophenol [23], kelthane [24], polychlorinated biphenyls (PCB) [25], polychlorinated dibenzo-*p*-dioxins [26], pentachlorophenol (PCP) [27], and 2,4,5-trichlorophenoxyacetic acid [28]. Results indicate that complete dechlorination (reduction reaction) of chlorinated compounds by the photocatalytic degradative method tends to be rather slow toward the mineralization of organic carbon atoms (oxidation reaction) to carbon dioxide. As an example, the TiO_2_-catalyzed photodecomposition of the herbicide 2,4-dichlorophenoxyacetic acid (2,4-D) in aqueous dispersions has been investigated extensively for several years [29,30,31,32]. This agricultural chemical is a widely used and highly toxic synthetic phytohormone (toxin) that the United States Environmental Protection Agency has classified as a suspected endocrine disruptor. Not surprisingly then that 2,4-D was chosen as a model pollutant in in examining the dismantling of chlorine-containing compounds [33].

**Figure 4:**
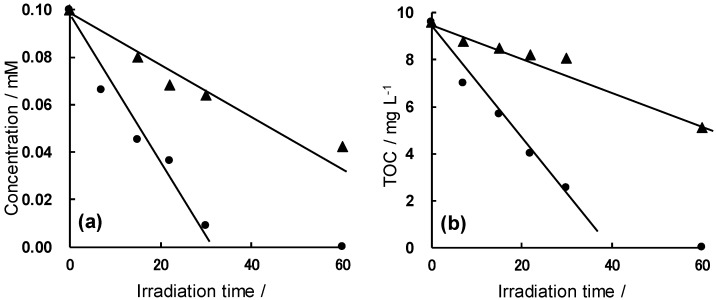
Disappearance of (**a**) UV absorption and (**b**) TOC in the photodegradation of 2,4-D by the UV and UV/MW methods. Reproduced from [33]. Copyright 2007 by Elsevier B.V.

The degradation of 2,4-D was monitored by the loss of UV absorption at 285 nm and loss of TOC using the UV and UV/MW methods for oxygen-purged 2,4-D/TiO_2_ dispersions. It was shown [33] that the decomposition rate of 2,4-D by the UV/MW method is accelerated when compared with the UV method (Figure 4a). Loss of TOC (Figure 4b) is consistent with the UV spectral observation. Detection and identification of the intermediates produced during the degradation of the 2,4-D substrate were carried out by electrospray mass spectral techniques (LC-MSD, Agilent Technologies Inc., Alpharetta, GA, USA).

**Scheme 2:**
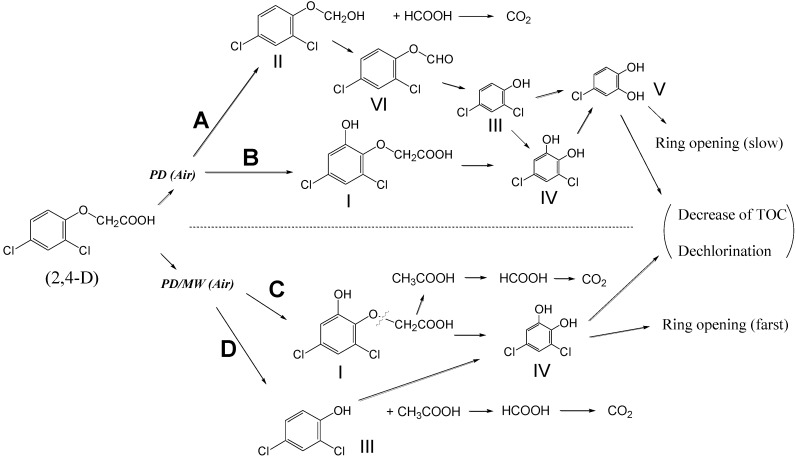
Proposed initial degradation mechanism of the photodegradation of 2,4-D by the UV and UV/MW methods. Reproduced from [33]. Copyright 2007 by Elsevier B.V.

Results from LC-MSD suggested the initial mechanistic steps (Scheme 2) for the photodegradation of 2,4-D by the UV and UV/MW methods [33]. The initial step in the UV method was taken as the cleavage of the C-C bond in the -CH_2_-COOH group in 2,4-D to yield formic acid and 2,4-dichlorophenoxymethanol (**II**) through addition of an •OH radical to the -^•^CH_2_ fragment (step A). Degradation of intermediate (**II**) then produced 2,4-dichlorophenoxyformaldehyde (**VI**), which on further irradiation yielded 2,4-dichlorophenol (**III**), followed by formation of 3,5-dichloro-1,2-benzenediol (**IV**) and 4-chloro-1,2-benzenediol (*i.e.*, 4-chlorocatechol; **V**). In step B, the 6**-**hydroxy-2,4-dichlorophenoxyacetic acid (**I**) was formed by attack of •OH radicals on the benzene ring, after which it led to the 3,5-dichloro-1,2-benzenediol species (**IV**) and to 4-chlorocatechol (**V**). For the UV/MW method, degradation of 2,4-D gave initially 6**-**hydroxy-2,4-dichlorophenoxyacetic acid (**I**) by the same mechanism as the UV method (step C). The acetic acid and formic acid intermediates were preceded by cleavage of the O-C bond in the O-CH_2_COOH fragment of 6**-**hydroxy-2,4-dichlorophenoxy acetic acid (**I**) and of 2,4-D (step D) to give the 2,4-dichlorophenol intermediate (**III**) and subsequently the 3,5-dichloro-1,2-benzenediol species (**IV**). The differences in these initial degradations are due to the initial cleavage position of the 2,4-D molecular structure. In other words, the C-C bond (83 kcal·mol^−1^) fragment was cleaved in the UV method, whereas the O-C bond (84 kcal·mol^−1^) was cleaved in the UV/MW method.

### 3.3. Degradation of 1,4-Dioxane in Aqueous TiO_2_ Dispersions

**Figure 5:**
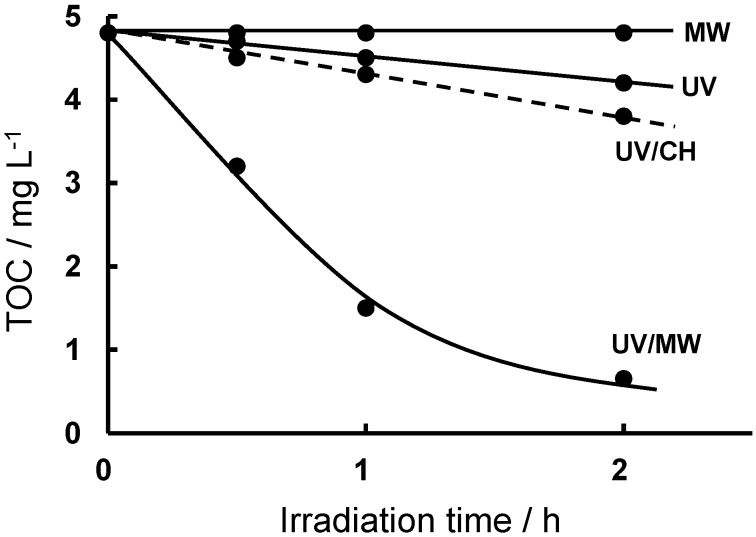
Decrease of TOC for the degradation of 1,4-dioxane. Reproduced from [34]. Copyright 2002 by the Japan Society of Color Material.

The degradation of an aqueous 1,4-dioxane solution with TiO_2_ was monitored by the decrease of TOC (Figure 5) [34]. The extent of decrease of TOC was 86% when the degradation was carried out by the UV/MW method for a 2-h irradiation period. By contrast, the decrease of TOC was only 13% and 21% by the UV and UV/CH methods, respectively. Under microwave irradiation alone, no decomposition of 1,4-dioxane occurred. The intermediates produced in the degradation of 1,4-dioxane were ascertained by LC-MS and HPLC-UV/Vis analyses [34]. The two oxygen atoms in the dioxane adsorbed on the TiO_2_ surface, following which peroxidation and/or hydroxylation events occurred with subsequent opening of the ring and ultimate formation of CO_2_ gas. The 1,2-diformyloxyethane (*i.e*., ethylene glycol diformate) and ethylene glycol intermediates were observed early in the decomposition by the UV/MW method, whereas they were generated gradually under UV and UV/CH irradiations. The degradation of aqueous 1,2-diformyloxyethane solution (0.10 mM) and aqueous ethylene glycol solution (0.10 mM) was also performed using the UV, UV/CH and UV/MW methods. The extent of TOC decrease for 1,2-diformyloxyethane was 2% (UV), 17% (UV/CH) and 79% (UV/MW), while the decrease of TOC for ethylene glycol was 17% (UV), 16.3% (UV/CH) and 76% (UV/MW). Clearly, the decomposition events of two of the intermediates identified in the degradation of 1,4-dioxane were also significant when carried out by the UV/MW method.

### 3.4. Non-Thermal Microwave Effect(s) in TiO_2_ Photoassisted Reactions

#### 3.4.1. Low Molecular Weight Organics

The degradation of many water contaminants by the UV/MW method is improved significantly when compared with the UV method. In addition to the decomposition of the initial contaminant, the decomposition of the resulting intermediates is also enhanced by the UV/MW method. To ascertain these inferences, studies examined the microwave specific (*i.e*., non-thermal) effects in relation to the chemical structure of low molecular weight compounds, whose photoassisted mineralization in the presence of titanium dioxide typically leads to formation of CO_2_ by an oxidation process via alcohol, aldehyde, and carboxylic acid intermediates (Reaction (1)) [35]. Delineating the effect of microwave radiation on these compounds provided an important base for clarifying the mechanistic features of the degradation and mineralization:

R-CH_3_ → R-CH_2_OH → R-CHO → R-COOH → RH + CO_2_ (1)

Accordingly, the tendency of water-soluble organics to be photomineralized under microwave and UV irradiation was examined to establish the mechanistic features using model substrates bearing alcoholic (methanol, ethanol 1-propanol, ethylene glycol and glycerin) and carbonyl functions (acetone, formic acid and acetic acid). Trends were seen from the decrease of the kinetics of loss of TOC with irradiation time [36]. The dynamics of the temporal losses of TOC on irradiation of the various dispersions by the UV, UV/MW and UV/CH methods are reported in Table 1.

**Table 1:** Kinetics (*k*) in the loss of TOC for 0–120 min of irradiation by the UV, UV/CH and UV/MW methods, except for methanol, formic acid and acetic acid that were irradiated only for 0–90 min. TH factor: increased rate by UV/CH method relative to UV; TH-MW factor: increased rate by UV/MW method relative to UV; and MW factor: increase in rate by the UV/MW method with respect to UV/CH. Reproduced from ref. [36]. Copyright 2007 by Elsevier B.V.

Compound	Mineralization Rate Constants, *k*_TOC_ (10^−3^ min^−1^)			Increase in Rates by Different Factors		
	UV	UV/CH	UV/MW	TH ^a^	TH-MW ^b^	MW ^c^
Methanol	1.0	9.1	25.3	9.1	25.3	2.8
Ethanol	0.9	2.7	3.4	3.0	3.8	1.3
1-Propanol	2.0	1.8	2.1	0.9	1.0	1.2
Ethylene glycol	1.6	2.2	9.0	1.4	5.6	4.1
Glycerin	1.8	6.3	11.9	3.5	6.6	1.9
Acetone	2.7	3.6	6.0	1.3	2.2	1.7
Formic acid	8.4	11.3	23.1	1.3	2.7	2.0
Acetic acid	3.9	6.1	20.5	1.6	5.3	3.4

**^a^** Thermal factor by comparing the *k*_TOC_ between the UV/CH relative to UV; **^b^** Thermal factor caused by the microwave irradiation on comparing the *k*_TOC_ obtained from the UV/MW method relative to UV; **^c^** Microwave factor from the comparison of *k*_TOC_’s from the UV/MW method relative to the UV/CH method.

*Methanol*: The rate of mineralization of methanol by the UV/MW method was nearly three times faster than by the UV/CH method, even though the temperatures at which the process occurred were identical. As well, the thermal effect was also not insignificant—the photoassisted mineralization of this substrate under conventional heating was nearly an order of magnitude faster; e.g., compare the UV/CH method with the UV method (the TH factor).

*Ethanol*: Adding one C atom to methanol caused no changes in the kinetics of the photoassisted mineralization of this substrate by the UV method. By contrast, the rates were significantly attenuated for the UV/CH and UV/MW by factors of ~3 and 3.8, respectively. The thermal factor also caused a threefold increase in the rate of mineralization, whereas the MW effect was somewhat subdued (factor of 1.3) indicating that the MW effect was somewhat limited relative to the TH factor.

*1-Propanol*: With the addition of two C atoms to methanol, both the TH and the MW factors no longer seemed to play a role in the mineralization of this substrate. The kinetics of mineralization were practically identical.

*Ethylene glycol*: Relative to methanol, this substrate contains an additional C atom as well as an additional OH function. The TH effect was considerably attenuated; *i.e.*, the rate of mineralization by the UV/CH method relative to the photoassisted UV method was just slightly faster. By contrast, the MW factor caused a significant fourfold increase in the rate of mineralization, whereas the combined TH–MW effect led to a nearly six-fold increase in the kinetics.

*Glycerin*: The structure of glycerin contains three C atoms and three OH functions, *i.e.*, one additional -CH_2_-OH unit with respect to ethylene glycol. The dynamics of the photoassisted mineralization of these two highly OH-loaded substrates by the UV method were experimentally similar. However, the TH factor increased the rates substantially by nearly a factor of 3.5 as evidenced by comparing the UV method with the UV/CH method. The MW factor also impacted on the mineralization of this substrate with the rate of mineralization by the UV/MW method being twofold faster than by the UV/CH method. Just as evidenced for ethylene glycol, the mineralization of glycerin and the corresponding TH and MW factors were somewhat attenuated relative to methanol, yet the photoassisted process (UV method) was enhanced, albeit to a small extent relative to methanol. Evidently, both the number of -CH_2_- units and -OH functions seem to play a role in either promoting or attenuating the degradation of the substrates with the assistance of the TH and MW effects.

*Acetone*: The TH effect and the MW effect had only a small influence on the dynamics of mineralization of acetone, whereas the rate of the photoassisted mineralization by the UV method was not very different from that of 1-propanol.

*Formic acid*: Of all the substrates examined, formic acid was mineralized faster by UV irradiation of the aqueous TiO_2_ suspension. Compared to methanol, the dynamics of mineralization by the UV/CH and UV/MW methods were rather similar. Regardless, while the TH factor was significantly different from that of methanol (1.3 *versus* 9.1; see column 5 of Table 2), the MW effect was only slightly smaller (2.0 *versus* 2.8).

*Acetic acid*: The rates of mineralization of acetic acid were about two times slower for both the UV and UV/CH methods relative to formic acid, whereas the rates for the UV/MW method were rather similar. However, even though the influence of the TH factors were similar for both formic and acetic acids, the MW effect was somewhat greater for the latter.

#### 3.4.2. Degradation of the Endocrine Disruptor BPA

**Figure 6:**
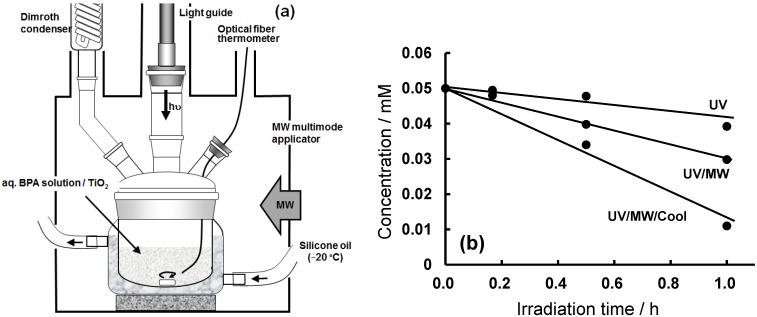
(**a**) Schematic illustration of the microwave-assisted photoreactor (MPR) coupled to a cooling system in the microwave multimode applicator; (**b**) Temporal decrease of the concentration of bisphenol-A (BPA: 0.050 mM) during its decomposition in aqueous media by photoassisted oxidation (UV), by the microwave-/photo-assisted oxidation (UV/MW) method, and by the integrated microwave-/photo-assisted degradation under cooling conditions (UV/MW/Cool). Reproduced from [38]. Copyright 2007 by Elsevier B.V.

In the degradation of various pollutants in wastewaters, the effect of microwaves manifested itself in the enhancement of the reaction dynamics of some aromatics that otherwise could not be obtained by conventional heating. In this regard, we examined the microwave-assisted photodegradation of bisphenol-A (BPA) in the presence of TiO_2_ at near-ambient temperature so as to unravel some details on the importance of the microwave non-thermal factor(s) [37]. BPA is one of many listed endocrine disruptors that tend to accumulate in the natural environment with serious consequential damage to the reproductive cycle in various animal species. To further delineate between the microwaves’ thermal and non-thermal factors, the degradation of BPA was later revisited [38] using an integrated microwave/ photoreactor (MPR) system that was equipped with a cooling system (see Figure 6a). The Pyrex reactor consisted of a double-layered structure with internal and external diameters of 75 and 100 mm, respectively. The silicone oil refrigerant was circulated through the inner part of this double layered structure using a circulation cooling apparatus. The silicone oil did not absorb the microwave radiation, and the temperature was maintained at −20 °C by the recirculation apparatus operating at the maximal flow rate possible. The course of the BPA degradation was followed using three experimental methods: (a) the UV/MW; (b) the UV/MW method under controlled ambient temperature with the cooling system (UV/MW/Cool); and (c) the photoassisted method alone (UV) mediated by TiO_2_. The temperatures of the solution by the UV and UV/MW/Cool methods were kept at 21 °C, whereas the temperature in the UV/MW method reached 85 °C after 1 h of irradiation.

The time course of the degradation of bisphenol-A by the UV, UV/MW and UV/MW/Cool methods is reported in Figure 6b. The relevant degradation dynamics subsequent to UV/MW irradiation were *ca.* twofold faster than for the UV method alone. The UV/MW/Cool method (*ca*. 21 °C) was twofold faster than for the degradation of BPA by the UV/MW method taking place at the higher temperature. The extent of degradation of BPA followed the order: UV/MW/Cool (78%) > UV/MW (42%) > UV (21%) after microwave and/or UV irradiation for 1 h. Clearly, the microwave-/photo- assisted degradation of BPA was most efficient when carried out at near-ambient temperature, under which the microwave-assisted photodegradation of BPA was not only due to a microwave thermal effect, but also to a significant non-thermal effect that might implicate the formation of hot spots on the TiO_2_ particle surface. The photodegradation was enhanced under constant ambient temperature. The non-thermal effect of the microwave radiation was also shown in the selective radical synthesis of 3-cyclohexyl-1-phenyl-1-butanone [39].

### 3.5. Increase in Radical Species on TiO_2_ under Microwave Irradiation

When a semiconductor particle is illuminated at wavelengths corresponding to photon energies equal to or larger than the bandgap energy, valence band electrons are excited to the conduction band and holes are produced in the valence band. Most of the electron-hole pairs produced recombine after some tens of picoseconds with the energy being released as emission of photons, phonons or both [40]. Coordination defects at the surface of the particle and defects within the particle lattice trap the remaining charges [41]. The holes trapped at the surface have a highly reactive oxidation potential and the electrons have a highly reactive reduction potential. Thus, photogenerated holes and electrons can induce reactions at the surface that, for historical reasons, are often referred to as photocatalytic reactions [42]. Titanium dioxide has a bandgap of 3.20 eV (anatase crystal), which corresponds to a wavelength of 387 nm. This means that electron-hole pairs are created when TiO_2_ is radiated with UV-light with wavelengths shorter than 387 nm (Reaction (2)). To the extent that not all the photogenerated electrons and holes recombine, some of the holes can migrate to the surface and react with surface-bound -OH groups and/or water molecules surrounding the particles that lead ultimately to the formation of hydroxyl radicals (Reaction (3)). Dissolved oxygen molecules react with conduction band electrons (e^−^) to yield superoxide radical anions (O_2_^−•^; Reaction (4)), which on protonation generate the hydroperoxy radicals ^•^OOH (Reaction (5)). Accordingly, the photooxidation of organic substrates with the UV/TiO_2_-driven photoassisted process (Reaction (6)) depends on the concentration of ^•^OH (and/or ^•^OOH) radicals produced by the photooxidation of surface hydroxyl groups and/or chemisorbed H_2_O.

TiO_2_ + h*v* → TiO_2_ (e^−^ + h^+^) → e^−^ + h^+^ (2)

h^+^ + ^−^OH_surf._ (and/or H_2_O) → ^•^OH (+ H^+^) (3)

e^−^_cb_ + O_2_ → O_2_^−•^ (4)

O_2_^−•^+ H^+^ → ^•^OOH (5)

^•^OH (or ^•^OOH) + organic pollutant → Oxidative products (6)

**Figure 7:**
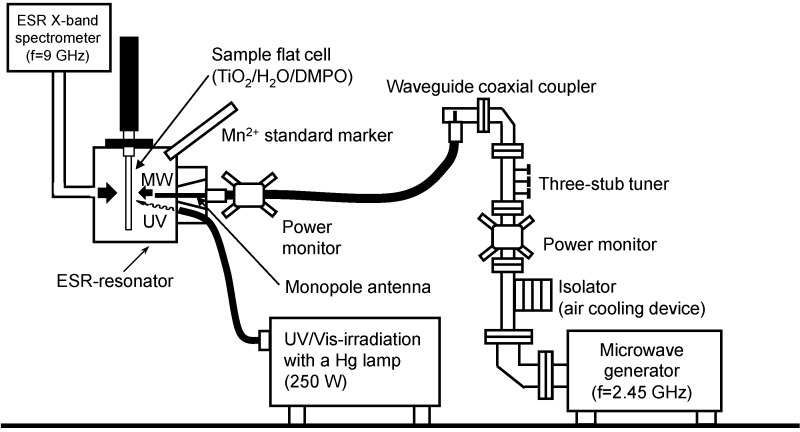
Setup used to generate ^•^OH radicals in water alone under MW irradiation, in an aqueous TiO_2_ dispersion by MW irradiation alone, and by the UV and UV/MW methods. Reproduced from [43]. Copyright 2003 by Elsevier B.V.

The possible enhancement of the photoactivity of metal-oxide specimens subsequent to being exposed to microwave radiation from the viewpoint of the amount of ^•^OH radicals generated was also investigated [43]. Formation of ^•^OH radicals during TiO_2_-assisted photooxidations that were driven simultaneously by UV light and microwave radiation was probed by electron spin resonance spectroscopy employing a novel setup in which the ESR sample (contained the DMPO spin-trap agent and TiO_2_ particles in aqueous media) was irradiated by both UV light and microwave radiation [11,43]. In this case, microwave radiation was produced using a magnetron microwave generator (frequency, 2.45 GHz), a three-stub tuner, a power monitor, and an isolator (Figure 7). The UV irradiation source was an Ushio 250-W mercury lamp; the emitted UV light irradiated the sample at an angle to the horizontal plane using a fiber optic light guide.

The number of ^•^OH radicals generated under various experimental conditions is summarized in Table 2. For P25 titania, the number of ^•^OH radicals produced by the UV/MW method was nearly 30% greater than the quantity generated by the UV method alone [43]. A fivefold increase in incident microwave power from 3 to 16 W led to a significant increase (*ca*. 40%) in the number of ^•^OH radicals. Such an increase was sufficient to increase the efficiency of the photooxidation of the organic pollutant in water.

**Table 2:** Number of DMPO-^•^OH spin adducts produced in the various heterogeneous systems under microwave irradiation, UV irradiation, and MW/UV irradiation relative to those formed in the rutile TiO_2_ specimen for the TiO_2_/H_2_O/MW heterogeneous system. Reproduced from [43]. Copyright 2003 by Elsevier B.V.

Methodology	P25	UV100	Anatase	Rutile
UV	182	45	110	110
UV/MW (3 W)	259	51	92	76
UV/MW (16 W)	369	-	-	-

For the UV100 sample, the increase in the number of ^•^OH radicals produced was only 10% greater on increasing the MW power five times. On the other hand, the number of ^•^OH radicals generated for the pristine anatase and rutile TiO_2_ samples decreased under microwave irradiation. The P25 specimen was clearly influenced by the microwaves and generated ^•^OH radicals efficiently under the influence of microwave effects. Therefore, the rate of decomposition was enhanced when P25 was used to decompose the wastewater sample by the UV/MW method. On the other hand, to the extent that the quantity of •OH radicals produced by the other TiO_2_ does not increase even when irradiated with microwaves, the rate of decomposition is not enhanced. To test this assertion, the photodegradation of aqueous 4-chlorophenol (4-CP) solution was examined using four different kinds of TiO_2_ particles [14,15]: Evonik P25 titania, Hombikat UV100 TiO_2_, Ishihara ST01 titanium dioxide (Ishihara Sangyo Kaisha Ltd., Nishi-ku, Osaka, Japan), and pristine anatase and rutile titania products (Wako Pure Chemicals Co. Ltd., Chuo-ku, Osaka, Japan). The physical properties of these various TiO_2_ specimens and the corresponding zero-order rates for the degradation of 0.025 mM 4-CP in aqueous media by the UV, UV/MW and UV/CH methods are reported in Table 3 [11].

**Table 3:** Physical properties of various TiO_2_ specimens and zero-order rates for the degradation of 4-chlorophenol (4-CP: 0.025 mM) using several methods: namely, the UV, UV/MW and UV/CH methods. Reproduced from [11]. Copyright 2009 by Copyright 2002 by the American Chemical Society.

Sample	Anatase (%)	Particle Size (nm)	Surface Area (m^2^·g^−1^)	Zero-Order Rates (10^−4^ mM·min^−1^)		
				UV	UV/MW ^a^	UV/CH ^a^
P25	82	33	52	1.0	2.5	1.7
UV100	100	10	323	0.6	1.4	1.5
Anatase	100	369	10	1.5	1.7	1.8
Rutile	0	263	16	0.02	0.05	0.17

**^a^** Reactions carried out under otherwise identical temperature conditions.

The microwave non-thermal effect(s) (UV/MW *versus* UV/CH) is clearly indicated in the Evonik P25 sample, which consists of well interwoven anatase (*ca*. 80%) and rutile (*ca*. 20%) crystallite forms; this sample is a well-known material that has manifested significant photoactivity in several photoreductive and photooxidative processes. The microwave thermal effect was observed in other TiO_2_ specimens. It is curious that the microwaves affect the P25 system and enhance its activity as deduced from our studies, from which we inferred to be due to some structural features (see below) inherent in this TiO_2_ system. Results are in agreement with the increase in the number of ^•^OH radicals produced by the microwave radiation.

Heat treatment of P25 titania and ST01 TiO_2_ in the presence of H_2_ caused the former to change color from white to light blue, while the latter changed from white to pale yellow with the colors being stable even after exposure of the samples to air oxygen. The change in the extent of lattice distortion of the heat/H_2_-treated ST01 specimen was two-times greater than for the P25 sample under otherwise identical conditions. The UV-visible absorption spectra of both sets of specimens revealed a broad unresolved band envelope above 400 nm that was attributed [44] to the formation of *F*-type color centers originating from oxygen vacancies [45]. The kinetics of photodegradation of 4-CP by these heat/H_2_-treated specimens were enhanced significantly under UV/MW irradiation (*ca.* three times) relative to irradiation by the UV/CH and UV methods. As well, the treated P25 sample was nearly 25% more efficient than the untreated sample, while the corresponding ST01 specimen was 85% more efficient. Such increased efficiency under UV/MW irradiation for the heat/H_2_-treated ST-01 specimen relative to the pristine ST01 sample was not due to a microwave thermal effect, but to a non-thermal effect of the microwaves impacting on the nanostructure of the metal-oxide samples [44].

## 4. Microwave Discharge Electrodeless Lamp

### 4.1. The Need for More Efficient UV Light Sources

It was shown in the preceding section that microwave radiation is effective in enhancing the photocatalytic activity of metal-oxide specimens. Accordingly, a treatment method that can treat larger quantities of pollutants in wastewaters is conceivable by a hybrid combination of the microwave technology and the photocatalytic technology. However, the light source can be a problem in the scale-up of microwave-assisted TiO_2_-photoassisted processes. The bandgap energy of anatase TiO_2_ is 3.20 eV, so that UV light less than 387 nm is necessary to activate this metal oxide. Generally, an electrode Hg lamp is used as the UV light source in Advanced Oxidation Processes. However, it is difficult to set up a typical mercury lamp in a microwave field from the viewpoint of the electrical discharge. In addition, it is difficult to open the window for the light beam to the microwave applicator because of possible leakages of the microwave radiation. Moreover, a small hole can only irradiate a small fraction of the dispersions. Accordingly, novel microwave-driven electrodeless lamps (MDELs) were developed by us (also photoreactors) and others as UV light sources to overcome many of the problems noted (see e.g., [46]).

### 4.2. Preparation of the MDEL Light Sources

**Figure 8:**
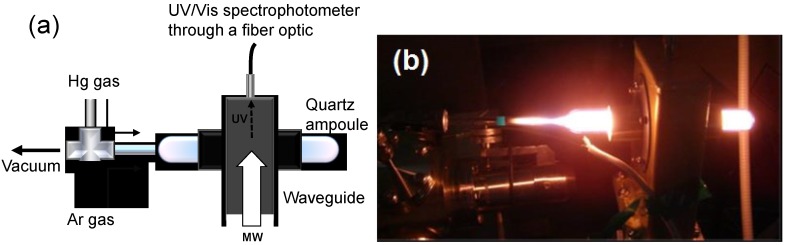
(**a**) Experimental setup for the examination of optimized conditions for a microwave discharge electrodeless lamp (MDEL); (**b**) photograph of nitrogen/argon mixed plasma light with the source of the MW radiation. Reproduced from [46]. Copyright 2009 by the Royal Society of Chemistry.

Optimized conditions to obtain the best gas mixture ratios and internal gas pressures in the MDEL devices were examined using the setup illustrated in Figure 8 [46]. A quartz ampoule connected to the vacuum system was positioned in the microwave waveguide. The size of the quartz ampoule (MDEL) was 145 mm (length) by 18 mm (diameter). The initial internal pressure in the ampoule was set at 10^−3^ Torr (or *ca*. 0.13 Pa) using a turbo molecular pump assisted by a rotary pump. Subsequently, mercury and argon gases (target gases) were introduced into the ampoule with the amount adequately adjusted by a mass controller. The pressure inside the ampoule was monitored with a capacitance manometer. The gas-mixture ratios were calculated from the volume ratio of each gas.

The UV-visible spectra of the emitted light plasma and the corresponding light intensities for each gas (and mixture) subjected to microwave irradiation were monitored through a fiber optic connected to a UV-Visible spectrophotometer (Figure 8a). The most suitable gas, gas-mixture ratio, and gas pressure for the MDEL device was determined using three criteria: (1) light intensity, (2) spectral pattern, and (3) self-ignition of the gases by MW irradiation alone. In one case, subsequent to evacuating the MDEL quartz envelope to 133 × 10^−7^ Pa, the system was purged with argon gas (133 × 10^−3^ Pa) after which a small quantity of liquid mercury was added. Figure 8b displays a photograph of the light emitted by the MDEL subsequent to MW irradiation.

**Figure 9:**
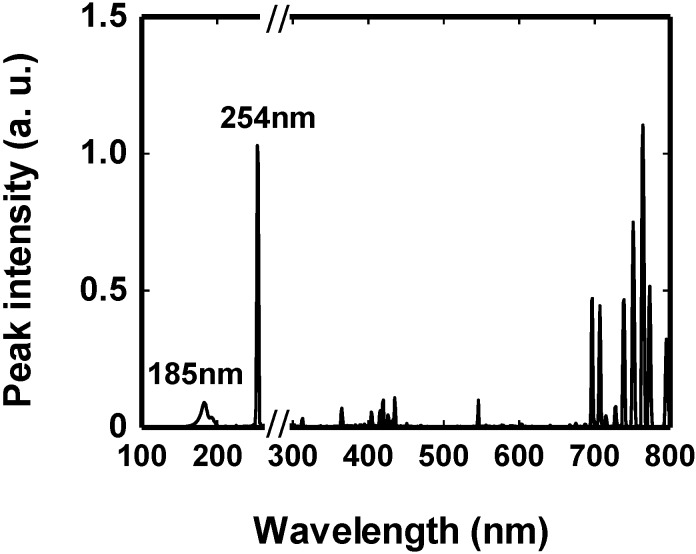
Vacuum-UV, UV, Visible and near-IR wavelengths emitted by the MDEL under microwave irradiation. Reproduced from [47]. Copyright 2009 by the Royal Society of Chemistry.

A 100-W microwave source was used to irradiate the MDEL device positioned in the multimode microwave applicator. The emitted vacuum UV light (VUV) was attenuated by the ozone generated from the oxidation of atmospheric oxygen. However, this decrease in irradiance could be minimized by purging the multimode applicator with nitrogen gas. The main peaks of the VUV and UV range were 185 nm and 254 nm, respectively (Figure 9) [47]. These peaks are similar to the wavelengths emitted by a traditional low-pressure mercury lamp.

### 4.3. Traditional Hg Lamp versus an MDEL Light Source in the Photodegradation of 2,4-D in Aqueous TiO_2_ Dispersions

An MDEL device of the same size as a domestic low-pressure mercury electrode lamp was used to examine the effect of the MDEL light source against a conventional Hg light source in the purification of a model wastewater sample containing the 2,4-D under otherwise identical power consumption [46]. The two UV electrode Hg lamps [dimensions: 10 cm (length) × 3 cm (diameter)] were located on top and bottom of a quartz pipe reactor. The TiO_2_/2,4-D aqueous dispersion was circulated using a peristaltic pump coupled to a cooling device to maintain the solution temperature at *ca.* 27 °C.

**Figure 10:**
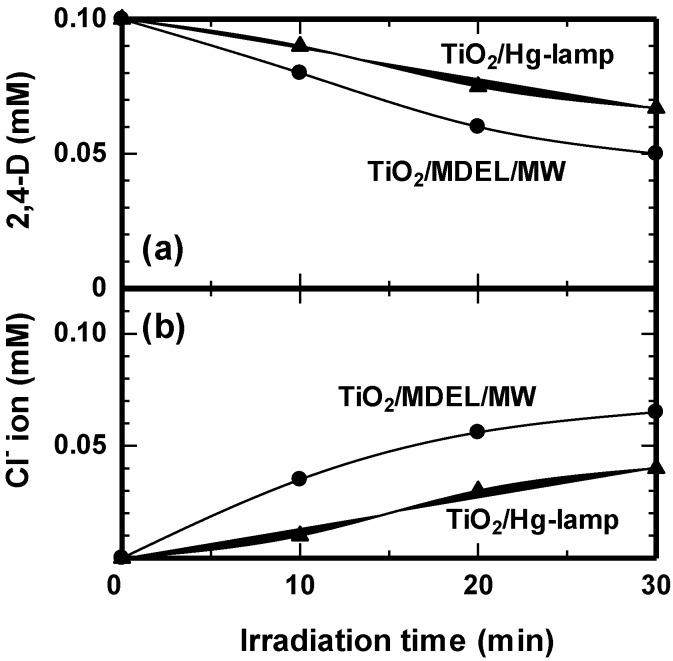
(**a**) Temporal variations in the concentration of 2,4-D during its degradation in aqueous solutions using MDEL and electrode Hg lamps; (**b**) formation of Cl^−^ ions during the dechlorination of the 2,4-D solution. Reproduced from [46]. Copyright 2009 by the Royal Society of Chemistry.

The degradation yields for 2,4-D using the electrode Hg lamp and the electrodeless Hg-filled lamp (MDEL) were 33% and 50%, respectively, after a 30-min irradiation period under otherwise identical conditions (Figure 10a) [46]. The corresponding levels of dechlorination (Figure 10b) of 2,4-D were 20% and 33%, respectively. The total electric power used to power the electrode and electrodeless Hg lamp systems was in both cases 150 W. However, the average light irradiance for the electrode Hg lamp was three times greater (*ca*. 12 mW·cm^−2^) than that of the electrodeless lamp (*ca*. 4 mW·cm^−2^). Clearly, the degradation of 2,4-D by the TiO_2_/MDEL/MW method, even at a smaller light irradiance, was faster than by the TiO_2_/electrode Hg-lamp method without microwaves but at higher light irradiance. Hence, microwave irradiation enhanced the overall degradation of 2,4-D and made up for the poor transparency of the dispersion and for the lower irradiance used.

### 4.4. Degradation of Perfluoroalkoxy Acids in Aqueous Media Using Small MDELs

Perfluoroalkylated pollutants have been detected globally in the wildlife [47], including in such remote areas as the Arctic [48]. Stable and chemically inert (C-F bond energy: 568 kJ·mol^−1^ or 5.882 eV, making it one of the thermodynamically strongest bond known), these perfluoroalkylated chemicals repel water and oil, reduce surface tension better than other surfactants, and work well under harsh conditions [49]. Perfluorooctanoic acid (PFOA, C_7_F_15_COOH) is one such pollutant that is used to make fluoropolymers that can release the PFOA precursor by transformation of some fluorinated telomeres. In 2006, eight major companies and the Environmental Protection Agency (USA) launched a stewardship program in which the industry was committed: (i) to reduce global manufacturing emissions and product content of perfluorooctanoic acid and related chemicals by 95 percent by 2010; and (ii) to work toward total elimination of emissions and product contents within the 2010–2015 period.

PFOA does not degrade naturally, and even with the use of advanced oxidation processes, its decomposition is rather difficult; as well, pyrolysis of PFOA necessitates relatively high temperatures [50]. Accordingly, a methodology that operates under milder conditions is most desirable for the decomposition and defluorination of PFOA and other perfluoroalkyls. Germane to this effort, Hori and coworkers reported the photodegradation of PFOA in aqueous media with and without a heteropolyacid photocatalyst (H_3_PW_12_O_40_•H_2_O) [51] or in the presence of persulfate (S_2_O_8_^2−^) [52]. In addition, the photoassisted defluorination and degradation of PFOA in aqueous media was also achieved by direct photolysis with 185-nm and/or 254-nm light from a low-pressure mercury lamp [53]. The extent of defluorination of PFOA attained by the latter method was 17% with the 185-nm VUV light after a 2-h irradiation period. In order to achieve the photodecomposition of perfluoroalkyls by the UV/MW method, we began to fabricate MDELs of a particular nature and design in our laboratory in Japan.

**Figure 11:**
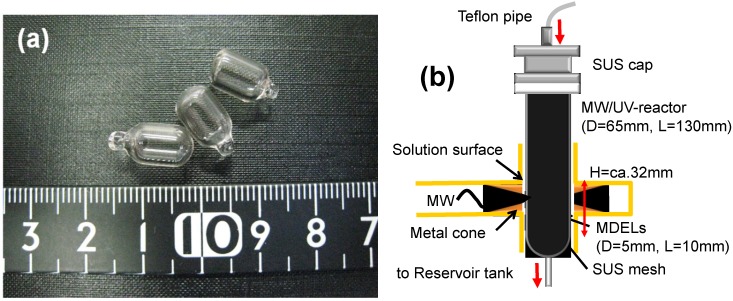
(**a**) Photograph of small MDELs and (**b**) the experimental setup of small MDEL device in a single mode microwave apparatus. Reproduced from [54]. Copyright 2011 by Elsevier B.V.

Small size microwave discharge granulated electrodeless lamps (MDELs) were constructed using vacuum-UV transparent synthetic quartz as the envelope and a mixture of Hg and Ar as the gas-fills [54]. Dimensions of the devices were 10 mm (length) by 5 mm (external diameter)—see Figure 11a. Subsequent to evacuating the MDEL quartz envelope to 133 × 10^−7^ Pa, the system was purged with argon gas (133 × 10^−3^ Pa) after which a small quantity of liquid mercury was added. Continuous microwave radiation was produced using a Hitachi Kyowa Engineering System microwave generator (frequency, 2.45 GHz; maximal power, 800 W), an isolator, a power monitor and a short-circuit plunger. The 300-mL air-equilibrated aqueous PFOA solutions were circulated with a peristaltic pump through the multipass MW/UV reactor (Figure 11b) containing the MDELs (20 pieces) at a flow rate of 600 mL·min^−1^. Note that no TiO_2_ was used in this experiment.

**Figure 12:**
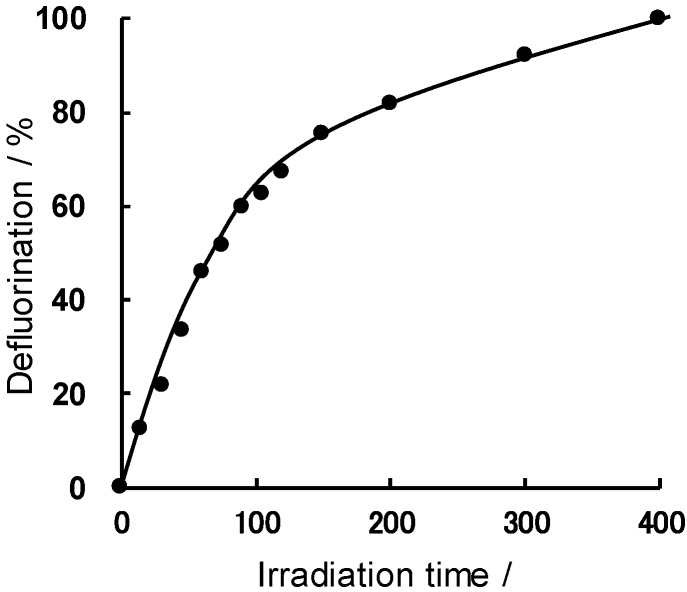
Time profiles of the extent of the photoassisted defluorination of perfluorooctanoic acid (PFOA) in aqueous solutions in the photoreactor containing the 20 MDELs systems. Reproduced from [54]. Copyright 2011 by Elsevier B.V.

Figure 12 illustrates the defluorination of perfluorooctanoic acid in aqueous media using the 20 MDELs as the light sources [54]. The extent of defluorination of PFOA was *ca*. 80% after 200 min, but reached 100% upon further irradiation for another 200 min.

### 4.5. Purification of Water Using TiO_2_-Coated MDEL Systems in Natural Disasters

**Figure 13:**
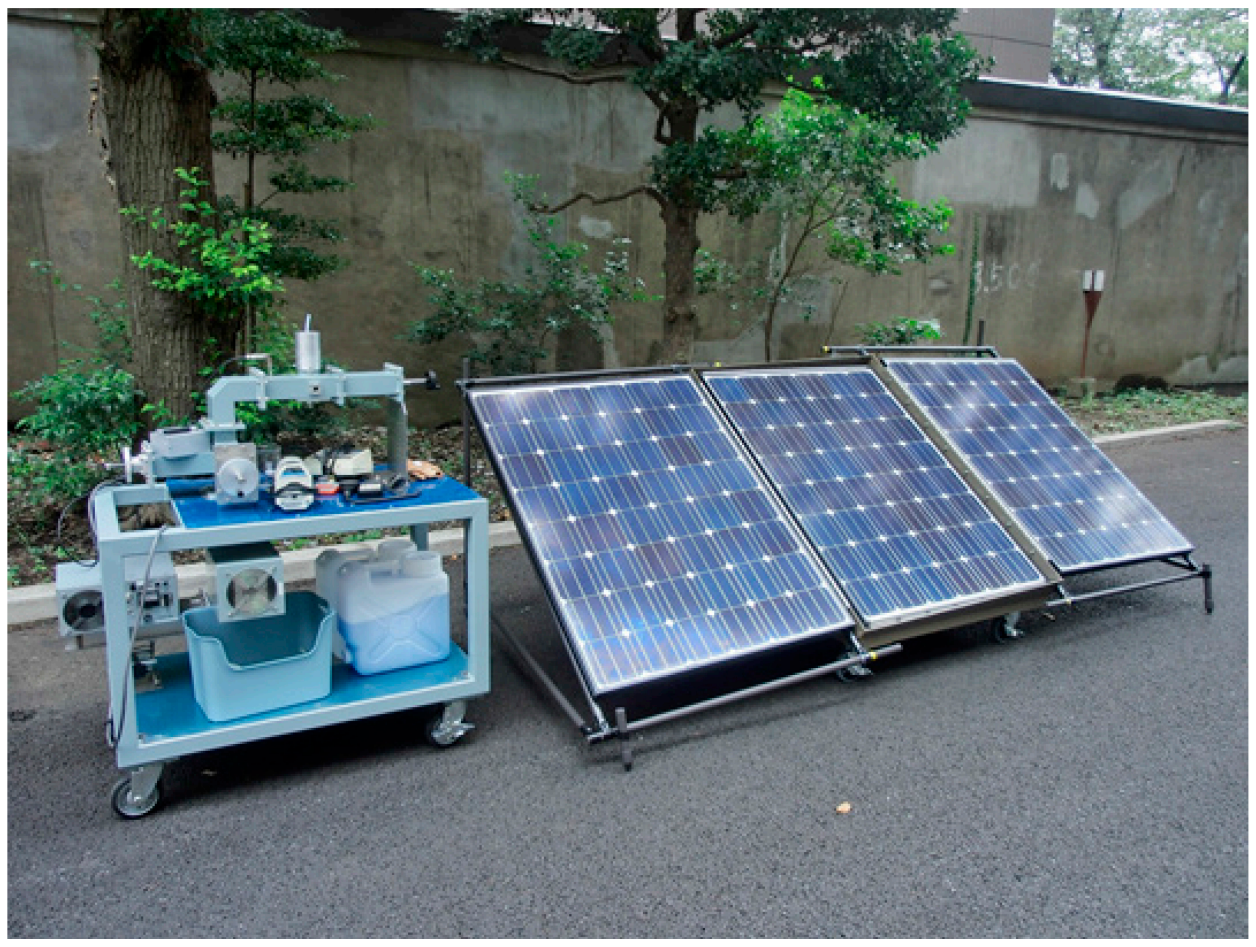
Water sterilization equipment used to sterilize rain water samples using the solar cells located on the right hand side of the photograph and the TiO_2_-coated MDELs (150 pieces) [55].

The great earthquake that occurred in eastern Japan on 11 March 2011 was soon followed by a huge tsunami; hundreds of houses collapsed either directly by the ground movement or else were destroyed during the subsequent tsunami. The demand for drinking water by sterilization of rain water increased immediately after the earthquake disaster. In connection with it, the needs of water for other uses (e.g., water to rinse off mud, water for toilets, *etc.*) also grew exponentially. Accordingly, we recently examined the possible sterilization of resurgent water in the earthquake-stricken area using MDELs and rain water collected in a pond (used as model rain water) using the setup illustrated in Figure 13 [55].

To the extent that an electric supply is typically unavailable in such areas after the earthquake and ensuing tsunami, the experimental setup also included a system of solar cells that were connected to the equipment so that water purification could be achieved. The sterilization equipment consisted of a reaction vessel that contained 150 pieces of bead-shaped MDELs (10 mm long by 5 mm in external diameter) positioned in the single mode microwave applicator. Sterilization was carried out by continuous introduction of the rain water sample from the reaction container located in the upper part of the setup. The equipment was so designed as to process natural water continuously on site. However, if used for long periods of time the surface of the various MDELs tends to become dirty. To overcome this issue, a thin layer of TiO_2_ nanoparticles was coated on the surface of the MDELs, such that the surface of the MDELs would remain clean by the well-known self-cleaning properties of titanium dioxide.

Preliminary experiments showed that a single pass is sufficient to sterilize more than 95% of the rain water (rate: 0.4 mL·min^−1^) through the reactor containing the TiO_2_-coated MDELs. Moreover, 100% killing of *Escherichia* *coli* (*E*. *coli*) was observed by a single pass through the reactor. In fact, sterilization of rain water was complete after only two passes through the reactor aided by an appropriate pump. Importantly, this equipment can also be used to process waters continuously that may have been contaminated with agricultural chemicals such as insecticides.

## 5. Summary Remarks

Some 15 years have passed since the discovery that led to the improvement in photocatalyst activity by the microwave radiation. The photocatalyst is a material that absorbs the energy of electromagnetic waves (UV light) and changes it into chemical energy. In this regard, microwave radiation also consists of electromagnetic waves. The notion of irradiating TiO_2_ with microwaves may appear strange at first because the photon energy (1 × 10^−5^ eV) of the microwaves of frequency 2.45 GHz is several orders of magnitude lower than the bandgap energy required (3.0‒3.2 eV) to activate the TiO_2_ semiconductor. Moreover, microwave radiation brings other effects to bear to a photocatalyst other than heat. Microwave non-thermal effects have been deduced to contribute significantly to the enhancement of a TiO_2_-photoassisted reaction, as it may affect both the surface and the crystalline structure of the metal oxide toward reactions taking place at the surface. The mechanism of the effect of microwave radiation on photocatalyzed reactions has been resolved gradually. The enhanced treatment of wastewaters through improvement in photocatalyst activity when exposed to microwave radiation is now a clear possibility. Coupling the microwave radiation with UV light in TiO_2_-photoassisted processes can contribute significantly to the treatment of wastewaters as a novel advanced oxidation technology (AOT).
